# Genome sequence of WestPM, a phage infecting *Microbacterium foliorum* isolated from beach environmental samples

**DOI:** 10.17912/micropub.biology.001395

**Published:** 2025-01-04

**Authors:** Charles J. West, Brittany C. Yencho, Andrew J. Brown, Conor R. Flannigan, Hui-Min Chung

**Affiliations:** 1 Department of Microbiology, University of Alabama at Birmingham, Birmingham, AL; 2 Department of Biology, University of West Florida, Pensacola, FL; 3 School of Veterinary Medicine, Louisiana State University, Baton Rouge, LA; 4 United States Environmental Protection Agency, Gulf Breeze, FL; 5 Department of Surgery, Duke University, Durham, NC

## Abstract

Bacteriophage WestPM is a siphoviral-like phage infecting *Microbacterium foliorum* isolated from environmental samples collected on Pensacola Beach, FL. The genome of this phage is 39,693 bp long and contains 59 predicted protein-coding genes and zero tRNA genes. Based on gene content similarity, WestPM is grouped in the actinobacteriophage EA11 subcluster.

**Figure 1. Morphological characterization of phage WestPM:**
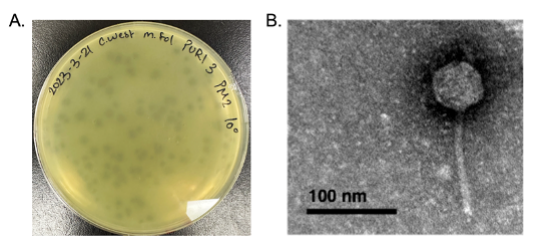
**A.** The plaque morphology of WestPM after three rounds of purification plated on PYCa medium. **B.** Negative staining transmission electron micrograph of *Microbacterium foliorum* phage WestPM revealing siphovirus morphology. Staining was performed using 1% uranyl acetate staining on Formvar-coated copper grids. Imaging was performed using a Hitachi H-7650 microscope operated at 75 kV and equipped with a 1kx1k CCD detection camera (Gatan 782). The magnification used was 200,000× with a pixel size of 5.18 Å at the level of the specimen. WestPM has a capsid with a diameter between 53-54 nm and a flexible tail of 110-118 nm (n = 2).

## Description

Bacteriophages are the most abundant biological entities on Earth and play critical roles in ecological balance, nutrient cycling, and microbial evolution (Chevallereau et al., 2021; Mayers et al., 2023) . Here we report a *Microbacterium foliorum* phage, WestPM, which was isolated from a mixture of coquina and surface sand collected from the shore of Pensacola Beach, Florida (GPS coordinates: 30.33073 N, 87.14191 W). For direct isolation, the sample was suspended in 14 mL PYCa (peptone-yeast extract-calcium) liquid medium for two days, and the supernatant was then filtered (0.22 μM). Viral particles in the filtrate were precipitated with PEG solution (PEG 1000), and the precipitate was then resuspended in 0.14 mL buffer before being plated in top agar with *Microbacterium foliorum* NRRL B-24224 (Russell et al., 2019) . After three days at 28°C, a representative ~1.5 mm-wide hazy plaque with a halo and irregular border was isolated, yielding WestPM ( **Figure 1A** ). WestPM was purified through three rounds of plating, and negative-staining transmission electron microscopy (TEM) revealed siphovirus morphology (Bradley, 1962; Zorawik et al., 2024) ( **Figure 1B** ).

DNA from WestPM was extracted from a phage lysate using the Norgen Biotek phage DNA isolation kit, prepared for Illumina sequencing using the NEB Ultra II Library Kit, and sequenced using an Illumina MiSeq (v3 reagents), generating 1,730,886 single-end 150-base reads, constituting 6541-fold coverage. After contig assembly through Newbler v2.9 and a check for completeness and genome termini through Consed v29, WestPM was determined to have a circularly permuted genome of 39,693 bp and possessing 63.9% GC content (Silva et al., 2013; Gordon et al., 1998; Russell and Hatfull, 2016) . The genome was annotated using DNA Master v5.23.6 (Pope and Jacobs-Sera, 2017) , PECAAN version 20230302 (Rinehart et al., 2016) , Glimmer 3.02b (Delcher et al., 2007) , GeneMark v2.5 (Besemer and Borodovsky, 2005) , BLAST using Actinobacteriophage and NCBI non-redundant protein databases (Sayers et al., 2021) , HHpred using PDB_mmCIF70, Pfam-A_v37, and NCBI Conserved Domains databases (Söding et al., 2005), DeepTMHMM 1.0.24 (Hallgren et al., 2022) , and Phamerator (Cresawn et al., 2011) , all using default parameters.

The annotation revealed 59 predicted genes, and 28 were assigned putative functions. Aragorn and tRNAscan-SE revealed the lack of tRNA genes within the genome (Laslett and Canback, 2004; Chan and Lowe, 2019) . Genes within the first half encode structural and assembly functions and are transcribed rightward, whereas the DNA metabolism functions and 16 genes of unknown function are encoded in the second half of the genome and are transcribed leftward. Based on gene content similarity (GCS) of at least 35% to phages in the Actinobacteriophage database, phagesDB (https://phagesdb.org), WestPM was assigned to phage subcluster EA11 (Gordon et al., 1998; Russell and Hatfull, 2016) , despite WestPM being isolated from beach sand and coquina samples while all other EA11 phages were isolated from soil samples. Notably, WestPM genes *38* , *49* , and *50* do not have homologs in other EA11 phages.

**Data Availability**

The sequence information of WestPM is available in GenBank with the accession number  PP978895 , and Sequence Read Archive (SRA) No.  SRX24338412 .
